# CalFitter 2.0: Leveraging the power of singular value decomposition to analyse protein thermostability

**DOI:** 10.1093/nar/gkac378

**Published:** 2022-05-17

**Authors:** Antonin Kunka, David Lacko, Jan Stourac, Jiri Damborsky, Zbynek Prokop, Stanislav Mazurenko

**Affiliations:** Loschmidt Laboratories, Department of Experimental Biology and RECETOX, Faculty of Science, Masaryk University, Brno, Czech Republic; International Centre for Clinical Research, St. Anne's University Hospital Brno, Brno, Czech Republic; Faculty of Information Technology, Brno University of Technology, Brno, Czech Republic; Loschmidt Laboratories, Department of Experimental Biology and RECETOX, Faculty of Science, Masaryk University, Brno, Czech Republic; International Centre for Clinical Research, St. Anne's University Hospital Brno, Brno, Czech Republic; Loschmidt Laboratories, Department of Experimental Biology and RECETOX, Faculty of Science, Masaryk University, Brno, Czech Republic; International Centre for Clinical Research, St. Anne's University Hospital Brno, Brno, Czech Republic; Loschmidt Laboratories, Department of Experimental Biology and RECETOX, Faculty of Science, Masaryk University, Brno, Czech Republic; International Centre for Clinical Research, St. Anne's University Hospital Brno, Brno, Czech Republic; Loschmidt Laboratories, Department of Experimental Biology and RECETOX, Faculty of Science, Masaryk University, Brno, Czech Republic; International Centre for Clinical Research, St. Anne's University Hospital Brno, Brno, Czech Republic

## Abstract

The importance of the quantitative description of protein unfolding and aggregation for the rational design of stability or understanding the molecular basis of protein misfolding diseases is well established. Protein thermostability is typically assessed by calorimetric or spectroscopic techniques that monitor different complementary signals during unfolding. The CalFitter webserver has already proved integral to deriving invaluable energy parameters by global data analysis. Here, we introduce CalFitter 2.0, which newly incorporates singular value decomposition (SVD) of multi-wavelength spectral datasets into the global fitting pipeline. Processed time- or temperature-evolved SVD components can now be fitted together with other experimental data types. Moreover, deconvoluted basis spectra provide spectral fingerprints of relevant macrostates populated during unfolding, which greatly enriches the information gains of the CalFitter output. The SVD analysis is fully automated in a highly interactive module, providing access to the results to users without any prior knowledge of the underlying mathematics. Additionally, a novel data uploading wizard has been implemented to facilitate rapid and easy uploading of multiple datasets. Together, the newly introduced changes significantly improve the user experience, making this software a unique, robust, and interactive platform for the analysis of protein thermal denaturation data. The webserver is freely accessible at https://loschmidt.chemi.muni.cz/calfitter.

## Graphical Abstract

**Graphical Abstract:**
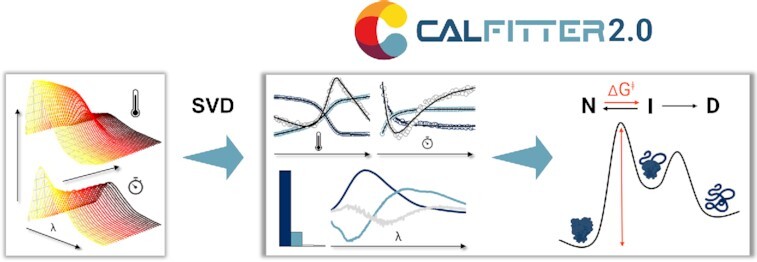
CalFitter 2.0 is a unique interactive platform for the analysis of protein thermal denaturation data, which newly incorporates singular value decomposition of multi-wavelength spectral datasets into the global fitting pipeline.

## INTRODUCTION

The thermal stability of proteins is imperative for their correct biological function, and its disruption often has devastating effects on the host organism. Protein instability leads to misfolding and aggregation that are associated with many severe human diseases, such as Alzheimer's, Parkinson's or Amyotrophic Lateral Sclerosis (1), and that gravely limit the efficient application of proteins in biotechnological, pharmaceutical, and other industries (2). Our general knowledge of the key structural and energetic basis of protein stability originates predominantly from the mutational unfolding studies (3,4). Although the framework for the proper analysis of thermodynamic and kinetic stability of proteins has a long history (5,6), experimental output from many stabilization studies is often limited to only a few empirical parameters, e.g. apparent melting temperatures (7). Considering the significant advancement in high-throughput biophysical techniques and a growing number of data-driven machine learning tools for protein stability prediction (8), the need for a robust, easy-to-use, and freely available platform for analysis of protein thermal denaturation data is therefore pressing.

To address this, we have previously developed the CalFitter webserver (9), which enables a global analysis of temperature-induced protein unfolding data measured with commonly used biophysical techniques, including differential scanning calorimetry (DSC), fluorescence, circular dichroism (CD), Fourier-transform infrared (FTIR) spectroscopies, and temperature jumps. The software integrates thirteen unique unfolding models, involving a various number of defined macrostates and different combinations of reversible or irreversible transitions between them. CalFitter 1.0 compiles the conventionally used reversible models as well as more complex partially or fully irreversible models collected from more recent literature (6,10). The former analyse the data based on the principles of equilibrium thermodynamics, whereas the latter treat the data from temperature scanning experiments as a dynamic process under kinetic control, sensitive to a particular scan rate, and integrate the equations describing the fractions of states numerically. The detailed mathematical description of these models can be found in the original publications (9,11). Experimental data can be interactively modelled based on the defined parameters, which allows users to easily test the validity of the selected model and make verifiable quantitative predictions about protein unfolding behavior. The output of the analysis is provided in an easily processible format, as physically relevant energy parameters derived based on the Eyring formalism of the transition state theory, e.g. Gibbs free energy differences (Δ*G*), which are being actively used as training data for recent machine learning stability predictors (12,13). To our best knowledge, it is the only tool that allows simultaneous fitting of data from temperature scanning experiments together with unfolding kinetics. The recent examples of CalFitter use include decoding the mechanism of domain-swapping of computationally stabilized haloalkane dehalogenase (14), explaining the kinetic stability of cold adapted subtilase (15), elucidating the aggregation propensity of polyketide cyclase (16), or study of dihydrofolate reductase evolution (17).

While CalFitter 1.0 has proved integral to the global data analysis of a wide range of experimental signals, recent technological advancements in massive data collection offer new opportunities for analysis yet to be fully exploited in the pipeline. Spectroscopic techniques are conveniently used to monitor protein unfolding due to their low sample requirements, moderate to high-throughput, and rich informational output. Earlier measurements were limited to an intensity change at a single wavelength (e.g. CD ellipticity) or the wavelength of the maximum intensity (fluorescence). However, such simple signals provide an incomplete picture of the unfolding process and are prone to misinterpretation (18). In contrast, recent technologies enable monitoring the entire protein spectra, which directly report on the local and global conformational changes during the unfolding. Yet this tremendous informational potential has not been fully exploited as it was not accompanied by the development of a suitable analytical toolbox for researchers without the advanced data analysis background.

In this work, we present a major update of the original CalFitter that addresses the current needs of the field in complete spectral data analysis using singular value decomposition (SVD). SVD is a powerful mathematical tool for data dimensionality reduction and has been exploited in several mechanistic studies of protein folding and unfolding using time-resolved fluorescence (19), small angle X-ray scattering (20–22), FTIR (23) and other advanced biophysical techniques (24,25). It is widely used for the detection of potential (un)folding intermediates due to its ability to extract spectral fingerprints of the protein states contributing to the overall signal (26–31). CalFitter 2.0 newly features (i) an easy upload of protein spectra recorded as a function of temperature (scanning experiments), time (kinetics), or other parameters (e.g. denaturant concentration, pH), (ii) the automated SVD analysis of these spectra, (iii) the interactive interface for dynamic visualization of the results and data pre-processing, (iv) the readily available export of the results in the excel format and (v) the global fitting of the SVD components from temperature scanning and unfolding kinetics experiments along with other signals, e.g. from DSC. The addition of the SVD analysis to the CalFitter pipeline greatly enhances the informational output of the software by providing spectral fingerprints of the relevant macrostates populated during protein unfolding. Additionally, based on the users’ feedback, we have completely reworked the data uploading procedure to accommodate various input file formats. The newly introduced changes significantly expand the applicability of the CalFitter 2.0 and make it a unique platform for global analysis of protein denaturation experiments.

## NOVEL FEATURES

**Figure 1.**
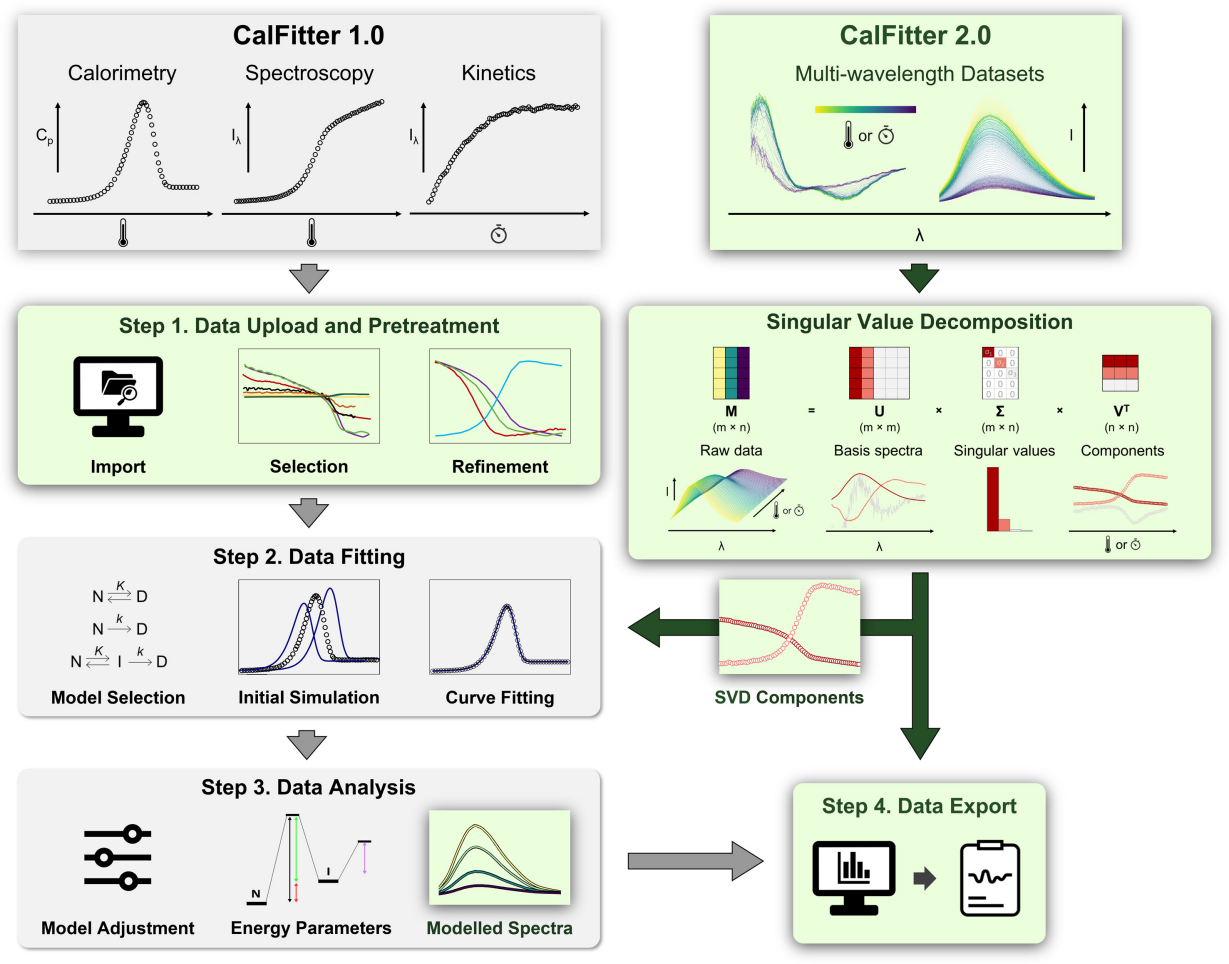
Overview of CalFitter workflow and newly introduced features. The features implemented into the original version 1.0 are shown in grey, while the novel features introduced into the version 2.0 are depicted in green. The details of individual steps and procedures are provided in the text or can be found in the original publication (9).

The original CalFitter has been described elsewhere (9), and its schematic overview, together with the novel functions introduced to the new version, are shown in Figure 1. The main feature of CalFitter 2.0 is the new SVD analysis module that is used as a data pre-processing step prior to the global fitting or as an independent tool for SVD analysis of virtually any multi-wavelength datasets. Another critical feature is a completely reworked uploading wizard supporting various input data formats and uploads from multiple files. Its interactive interface allows the quick and intuitive selection, labelling, visualization, and pre-processing of the input datasets. We provide a detailed description of the uploading wizard in the help section of the webserver https://loschmidt.chemi.muni.cz/calfitter/?action=help.

## SVD ANALYSIS

**Figure 2.**
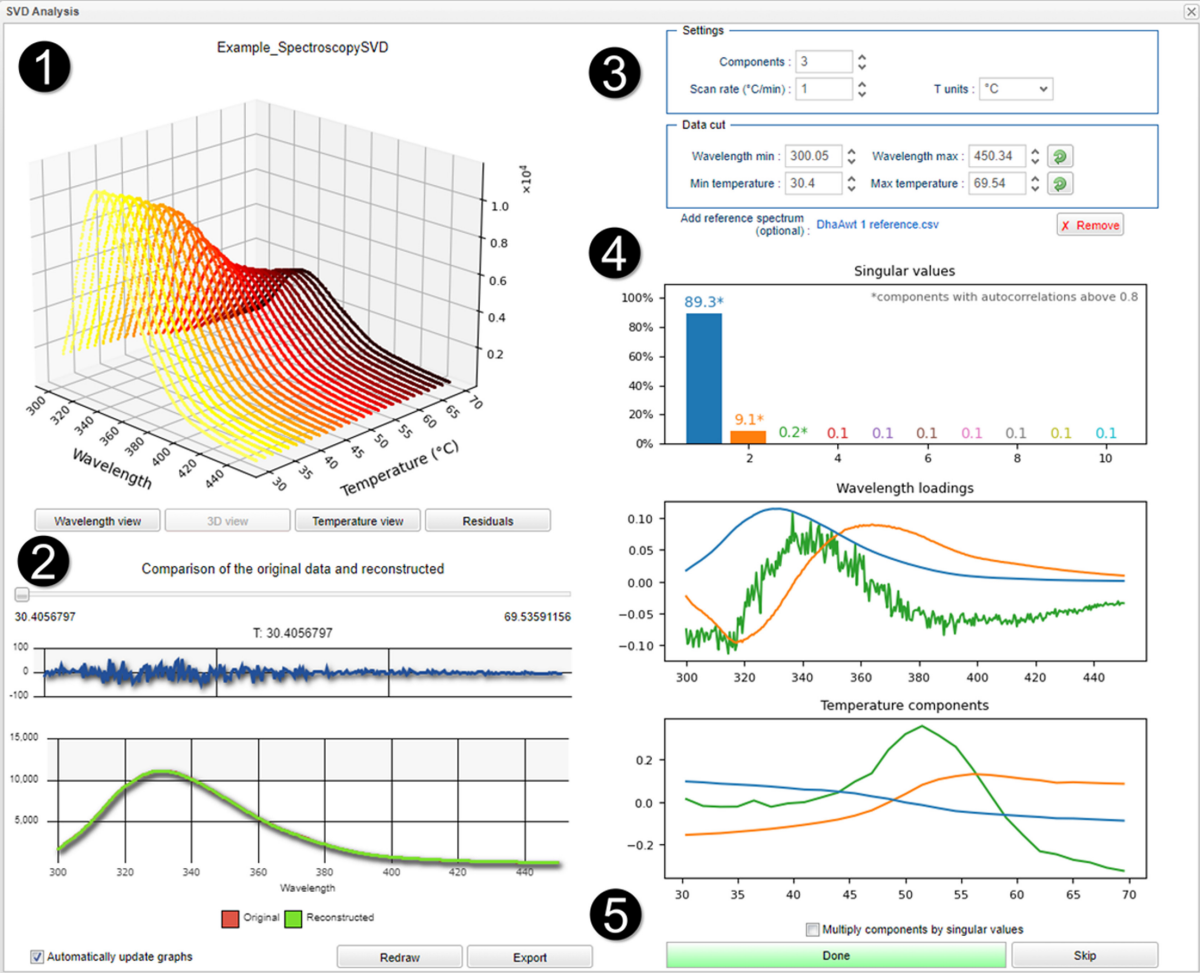
Interactive CalFitter 2.0 SVD analysis interface. The interface sections include (1) raw data visualization, (2) spectral reconstruction, (3) experimental parameter specification and data range settings, (4) SVD analysis results graphs and (5) export and upload options. The example data depict the thermal denaturation of the haloalkane dehalogenase DhaA (UniProt ID: P0A3G2), measured by following the changes in intrinsic protein fluorescence at the heating rate of 1°C/min. The asterisks in the Singular values plot indicate that the first three components have the autocorrelations of both the wavelength loadings and amplitude vectors above 0.8.

The input to the singular value decomposition consists of multi-wavelength data organized in a rectangular *m* × *n* matrix in which the *m* rows represent the wavelengths, and the *n* columns represent the experimental points, e.g. spectra at different times or temperatures. The SVD is a factorization of the original matrix to three matrices in the form of *U*Σ*V*^T^ (Figure 1), where the columns of the U are the left singular vectors (basis spectra), Σ contains singular values (component amplitudes) on its diagonal, and the rows of the *V*^T^ are the right singular vectors (time or temperature components). The detailed mathematical description of the algorithm procedure, together with the results of its thorough validation, can be found in the Supplementary Data (Table S1). CalFitter 2.0 performs the SVD automatically upon the data upload and displays the results in an interactive interface (Figure 2).

The graphical representation of the SVD results is displayed on the right side of the interface (section 4 in Figure 2). The first ten normalized singular values are shown in the bar graph. While their total number corresponds to the number of wavelengths or experimental points of the original dataset (whichever is lower, i.e. min{*m,n*}), generally fewer than ten components are sufficient to confidently reconstruct the original data. The numbers in the bar graph translate to the variation within the dataset that is explained by the respective component (>98% of data variation is sufficiently explained by the first two components in the example in Figure 2). The determination of the correct number of significant components for further analysis must be done with great care so that they truly represent all important features of the original dataset. Usually, the visual inspection of the shape of the basis spectra and regular patterns of the SVD components is the most robust yet subjective criterion. Alternatively, one can apply a cumulative threshold for the explained variation in the data (e.g. 98%) and keep only the components that are above it. Several statistical measures can also aid in the decision. The autocorrelations of each component basis spectrum and amplitude vector have been shown to provide practical guidance in determining whether a particular component captures the meaningful signal or noise in the data (32,33). To aid the users in the selection, CalFitter 2.0 marks the components whose autocorrelation coefficients are above the 0.8 threshold by an asterisk in the Singular values graph. Their exact values for each component are provided in the export Excel file, and a detailed description of how these values are calculated can be found in the Supplementary Data. In general, the explained variation and the autocorrelation methods can be applied when a more rigorous quantitative assessment of the SVD results needs to be carried out. However, the visual inspection of the components and their singular values usually suffices to make the decision.

Basis spectra (wavelength loadings) are depicted in the middle panel of section 4 in Figure 2. Typically, only the first few of them correspond to the meaningful signal components, while the others represent the experimental noise (Figures S1 and S3). The number of components to display and use in the original data reconstruction and subsequent global data analysis can be changed in the settings section (section 3 in Figure 2). The unique feature of CalFitter 2.0 is the possibility to assign the basis spectrum of the first component to that of a known protein state (typically native state, but others can be used) by uploading its spectrum as a reference. This increases the interpretability and biological relevance of the SVD results by providing spectral fingerprints of other relevant protein species populated during unfolding. The SVD is automatically recalculated when the reference spectrum is uploaded.

Finally, the changes of the component amplitudes with time (Kinetics SVD) or temperature (Spectroscopy SVD) are shown in the bottom right graph. These progress curves report on the evolution of the components throughout the course of the experiment and can be subjected to the subsequent global analysis of denaturation experiments. These curves are fully integrated into the workflow of CalFitter 1.0, i.e. they are modelled and fitted analogously and alongside the other two-dimensional signals such as calorimetry, spectroscopy, and kinetics (see Global Fitting of SVD Datasets).

The SVD analysis is fully automated in CalFitter 2.0, and all graphs dynamically change in response to the changes in parameter settings or dataset range. Spectral reconstruction of the raw data based on the selected number of components can be investigated by moving the slider below the raw data display on the left-hand side of the interface (section 2 in Figure 2). Export of the SVD results to an excel file is readily available. In principle, the SVD module can be used to analyse any type of multi-wavelength data regardless of the dynamic component (e.g. pH, denaturant, salts). However, the use of the SVD components in subsequent global fitting is restricted to the time- or temperature-dependent multi-wavelength spectral datasets collected at fixed temperatures or scan rates, respectively.

## GLOBAL FITTING OF SVD DATASETS

The global analysis interface and general procedure of CalFitter have not changed significantly since the first version, and their description is provided in the original publication (9). The *Data pre-treatment* panel of the global analysis interface has been newly expanded by two additional tabs devoted to *Spectroscopy SVD* and *Kinetics SVD* datasets. The data treatment options are identical to the corresponding non-SVD data types, i.e. specification of temperature range and normalization is possible for spectroscopy data, and collation and endpoint selection for kinetics data. The SVD components available for fitting are restricted to those selected during the SVD analysis. We recommend that only the non-noise SVD components are used for the global analysis to avoid overfitting. These are fitted similarly to other spectroscopic signals using a weighting procedure based on the number of points to ensure the balanced contribution of datasets to the penalty function of the fitting procedure (9).

**Figure 3.**
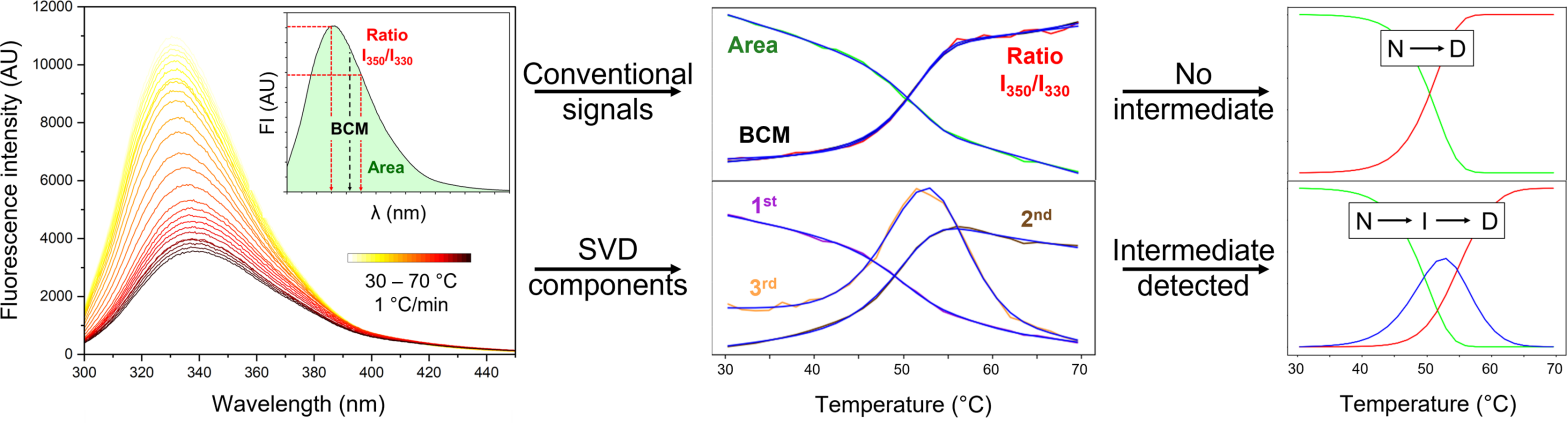
Differences between global fitting of single wavelength datasets and SVD components. Left: Thermal unfolding of DhaA monitored by fluorescence spectroscopy at the 1°C/min scan rate. Inset: The derivation of the conventional signals commonly used for representation of the changes in fluorescence spectra during protein denaturation: the ratio of fluorescence intensities at 350 nm and 330 nm (*I*_350_/*I*_330_), the barycentric mean of the spectrum (BCM, also referred to as the average emission wavelength), or integrated area of the spectrum. Middle: Comparison of the stability curves derived using the normalized single variables, and the normalized amplitude changes of the first three SVD components calculated from the dataset (corresponding to the SVD analysis shown in Figure 2). Right: The fraction of the states calculated from the global fit (blue lines in the middle panel) of the two-dimensional variables and the SVD components to the two- and three-state unfolding models, respectively. N, native; I, intermediate; D, denatured.

The SVD output is a more accurate and unbiased representation of the original dataset compared to the conventional two-dimensional signals, e.g. using intensity change at fixed wavelengths or the area under the spectrum. The SVD preserves the informational content of the raw data while reducing its dimensionality. In contrast, the selection of an appropriate 2D signal reflecting the spectral changes during denaturation is made empirically, typically by comparing differences between spectra of the native and denatured states. As a result, these signals are often insensitive to potential intermediates that can be only scarcely populated during unfolding. For example, in Figure 3, we show the analysis of the unfolding of haloalkane dehalogenase DhaA monitored by fluorescence spectroscopy. The denaturation curves constructed from the conventionally used signals reporting on the redshift of the fluorescence maximum (the ratio of intensities at 350 nm and 350 nm, barycentric mean – BCM), or overall intensity (the area under the spectrum) both show a single transition with the overlapping midpoint temperature around 50°C, which can be fitted into a simple two-state unfolding model (Figure S4A). However, the SVD of the raw data results in three significant components, indicating the presence of an intermediate state. A closer inspection reveals that while the first two components reflect the changes captured by the two-dimensional signals, the third component, albeit less significant in explained variance (∼0.2%), has the autocorrelations above 0.8 and shows two distinct transitions. In fact, all the three components fit well to the models involving an intermediate state (Figures S4B, C). Since the singular value of the third component is low, we confirmed the presence of the intermediate by an additional measurement using another experimental technique. In our model case, DSC thermograms consisted of two transitions and were fitted alongside the SVD components to the three-state partially unfolding model (Figure S5).

Another major advantage of fitting SVD components over the conventional two-dimensional signals is the ability to back-calculate the full spectra based on the modelled parameters and compare them to the original data (Figure S6). The reconstruction of the original spectra is carried out by a linear product of the modelled SVD components and the original SVD basis spectra. The visual comparison of data reconstructed from the fitting of a different number of components, therefore, provides additional means for model validation, identification of potential deviations from the data, and evaluation criteria of potential data overfitting. In the example case study, the first two components fit well to the two-state model, but the reconstructed spectra deviate from the raw data (Figure S4A). Only the global fit of all three components to the three-state model provides satisfactory spectral reconstruction (Figures S4B, C). The detailed description of the global analysis of multiple thermal denaturation experiments, including different SVD datasets, is shown and discussed in detail in the Supplementary Data (Section Use case Figure S1–S6).

## DATA INPUT AND OUTPUT

We have completely reworked the uploading procedure of the non-SVD datasets based on user feedback to improve its flexibility and user-friendliness. The software newly supports a variety of input formats, including Excel .xlsx files with multiple spreadsheets and fewer requirements on the data organisation. The new uploading wizard enables numerous interactive data pre-treatment options, including dataset visualisation, removal, column designation and parameter specification. Simultaneous upload and quick processing of multiple SVD datasets from single or different Excel files is also supported. At the same time, the input procedure is backward-compatible, i.e. when the legacy data format is recognised, the uploading wizard automatically prefills all the parameters accordingly. Similarly, the results of the SVD and global analyses can be easily exported at different stages of the process. Output datasets are organized logically in multiple spreadsheets within a single Excel .xlsx file. A detailed step-by-step description of the uploading interfaces and exporting options is provided in the help section of the webserver, which can be found at https://loschmidt.chemi.muni.cz/calfitter/?action=help. Altogether, all data manipulation steps have been significantly improved to ensure fast and intuitive application of the CalFitter and promote its wider use in the scientific community.

## CONCLUSIONS AND OUTLOOK

In summary, the main new features and improvements introduced to CalFitter 2.0 include: (i) automated SVD of multi-wavelength data in an interactive interface, (ii) global fitting of time- and temperature-dependent SVD components with other types of data from protein thermal denaturation experiments, (iii) spectral reconstruction of data based on the modelled parameters, (iv) the option of uploading a reference spectrum of a known protein state in the SVD analysis, (v) the improved data uploading procedure from multiple data formats and (vi) the flexible and intuitive uploading wizard with variety of data pre-treatment options. The implementation of SVD into CalFitter 2.0 provides an extra resolution to its informational output. We hope that this unique combination of the two complex mathematical analyses, i.e. SVD and global fitting, in the single, highly interactive, and freely available platform greatly diminishes the expertise requirements for their routine application. CalFitter strives to be the gold standard for the analysis of thermal denaturation experiments, providing invaluable quantitative parameters of protein thermostability, which are crucial for the development of efficient and accurate protein engineering tools.

In the future, we plan to introduce new algorithms for automatic initialization of model parameter values based on the input data, which will make the fitting procedure much easier, especially for first-time users. Moreover, we intend to extend the analytical scope of CalFitter by introducing models involving temperature-induced concentration-dependent aggregation and an entirely new module for analysis of chemical denaturation experiments. This will allow users to analyse the effects of various protein perturbants (e.g. solvents, additives, pH) on protein energetics in combination with temperature and extract valuable thermodynamic and kinetic parameters from multi-dimensional energy landscapes, which is particularly relevant for studying complex phenomena, e.g. cold denaturation. Another promising direction is an interactive model editor that will enable users to schematically draw any unfolding scenario, for which the software will automatically derive the underlying mathematical description and respective parameters. These changes will make CalFitter the ultimate one-stop shop for the analysis of protein stability.

## DATA AVAILABILITY

CalFitter 2.0 is freely available at https://loschmidt.chemi.muni.cz/calfitter/. The datasets used for the case study and numerical validation are provided in the Supplementary data.

## Supplementary Material

gkac378_Supplemental_Files
