# Supplementary material for: CalFitter 2.0: Leveraging the power of singular value decomposition to analyse protein thermostability
Source: Nucleic Acids Res. 2022 May 17;50(W1):W145–51. doi: 10.1093/nar/gkac378 (PMC9252748; doi:10.1093/nar/gkac378)
Supplement: gkac378_Supplemental_Files [file gkac378_supplemental_files.zip › CalFitter 2_SI_REVISED_final_.pdf]

Supplementary Data

CalFitter 2.0: Leveraging the Power of Singular Value Decomposition to Analyse Protein Thermostability

Antonin Kunka[1,2¶], David Lacko[3¶], Jan Stourac[1,2], Jiri Damborsky[1,2], Zbynek Prokop[1,2*], Stanislav Mazurenko[1*]

[1] Loschmidt Laboratories, Department of Experimental Biology and RECETOX, Faculty of Science, Masaryk University, Brno, Czech Republic
[2] International Centre for Clinical Research, St. Anne's University Hospital Brno, Brno, Czech Republic
[3] Faculty of Information Technology, Brno University of Technology, Brno, Czech Republic

* To whom correspondence should be addressed. Tel: +420 549 4930 41; Fax: +420 549 4962 03; Email: mazurenko@mail.muni.cz:

Correspondence may also be addressed to zbynek@chemi.muni.cz

¶The authors wish it to be known that, in their opinion, the first 2 authors should be regarded as joint First Authors.

Singular value decomposition (SVD)

This section covers the mathematical aspects of the SVD implementation and validation in CalFitter 2.0.

Basic analysis

SVD is a common matrix factorization technique, which allows the decomposition of the input *m* × *n* matrix M into the product of three matrices: the *m* × *m* matrix of basis spectra U, the *m* × *n* diagonal matrix of singular values Σ, and the *n* × *n* matrix of signal components V. Mathematically, it is stated as

$$M = U\Sigma V^T = \sum_{i=1}^{r} \sigma_i u_i v_i^T, \quad (1)$$

where $r \leq \min\{n, m\}$ is the rank of M, σ_i are nonnegative singular values, typically in descending order, u_i are basis spectra, and v_i are signal components. The columns u_i and v_i of matrices U and V, respectively, form two orthonormal sets. In other words, the sum in equation 1 decomposes the input matrix M into orthogonal contributions of r terms. Multiplying equation 1 by u_i^T on the left and transposing both sides of the equation, we obtain another interpretation of SVD:

$$M^T u_i = \sigma_i v_i \quad (2)$$

Therefore, each u_i can be seen as a set of weights (positive, negative, or zero) and $\sigma_i v_i$ is the corresponding weighted sum of rows of the matrix M (v_i is the normalized signal and σ_i is the scaling factor). In protein unfolding experiments, *m* typically stands for the number of wavelengths, and *n* corresponds to the number of data points, e.g., spectroscopic measurements at different time or temperature points. Therefore, each row of the matrix M is the amplitude of the signal at a fixed wavelength, and $\sigma_i v_i$ is a vector of weighted sums of these signals.

While SVD is not unique, e.g., multiplying both u_i and v_i by -1 will give the same formulas as equations 1 and 2, it allows exploiting the power of the best approximation of the matrix M. The Eckart–Young–Mirsky theorem states that keeping just the first k terms in equation 1 ($0 < k < r$) gives the best k-rank approximation of matrix M as judged by the Frobenius norm (1). Therefore, if the goal is to select just a few weighted combinations of different wavelengths for further analysis, keeping the first k columns of U as the sets of weights is optimal in a linear algebra sense. In the case of CalFitter 2.0, this translates to selecting the first k signals $\sigma_i v_i$ for further fitting.

CalFitter 2.0 allows fitting the selected signal components with a user-defined model of protein unfolding. For each component $\sigma_i v_i$ selected for fitting, a simulated signal s_i is modelled based on the current set of parameters, and these parameters are then updated iteratively to minimize the difference between $\sigma_i v_i$ and s_i (see the original article (2) for more details on the fitting procedure). Apart from the visual inspection of the graph showing the final s_i and $\sigma_i v_i$ or the corresponding residuals, the user can evaluate the quality of the fit at the level of original data, i.e., the matrix M. For this, the matrix corresponding to the simulated signals s_i is reconstructed based on equation 1 with $\sigma_i v_i$ replaced by s_i:

$$\widehat{M} = US^T, \tag{3}$$

where S is the matrix of s_i in columns.

Finally, the autocorrelation coefficients provide practical quantitative measure of the component noise and can be used in addition to the visual inspection for the specification of the correct number of components representing the raw data. They are calculated as follows:

$$C(U_i) = \sum_{j=1}^{m-1} u_{j,i}\, u_{j+1,i} \quad C(V_i) = \sum_{j=1}^{n-1} v_{j,i}\, v_{j+1,i} \tag{4}$$

Here, $C(U_i)$ is the autocorrelation coefficient of the *i*-th basis spectrum, $u_{j,i}$ and $u_{j+1,i}$ represent the *j*-th and the *(j+1)*-th elements of the *i*-th column of the matrix U, respectively. Similarly, $C(V_i)$ is the autocorrelation coefficient of the *i*-th temperature component, calculated correspondingly from the elements in the columns of matrix V.

Reference spectrum

While the first k basis spectra are optimal from the point of view of linear algebra, the SVD analysis alone does not consider any additional information a researcher might have from the experiments. One possibility to incorporate this prior knowledge into the SVD results is by fixing one set of row weights to those of the spectrum of a known protein state. For example, the unfolding experiments start at conditions that favor the native state, so the corresponding spectrum is therefore easily accessible and can be accounted for in the SVD analysis when uploaded as a reference spectrum.

When the reference spectrum is uploaded to CalFitter 2.0, u_1 – the first column of U – is fixed to its normalized form, i.e., rescaled to the norm of 1. Then, using equations 1 and 2, its contribution to the original data matrix M is subtracted, yielding the matrix of residuals M':

$$M' = M - \sigma_1 u_1 v_1^T = M - u_1 u_1^T M. \quad (5)$$

Performing the SVD on the residual matrix $M' = U'\Sigma' V'^T$ provides the remaining significant columns of the matrix U: $u_{i+1} = u_i'$, $i = 1, 2, \ldots rank(M')$. The remaining $m - rank(M') - 1$ columns do not contribute to the final decomposition and can be neglected, e.g. filled with zeros.

Notice that although u_1 does not have to lie in the space of the first r basis spectra of the SVD of the original matrix M, the rank of M' can still be equal to that of M, potentially adding one more nonzero term to equations 1 and 2. At the same time, since U' has orthonormal columns and $u_1^T M' = 0$ (equation 5), the matrix U will also have orthonormal columns. And once the matrix U is constructed, the corresponding r or $r + 1$ components of matrices Σ and V can be derived from equation 2 as:

$$v_i = \frac{1}{\|M^T u_i\|} M^T u_i, \quad \sigma_i = \|M^T u_i\|. \quad (6)$$

Here $\|\cdot\|$ denotes the Euclidian norm of a vector.

Implementation

The web user interface and backend are written in Java 8 and Smart GWT library 6.0. The web server runs the calculations and graph depictions by calling the Python scripts. The computational part of CalFitter is implemented in the Python 3, primarily using NumPy 1.19.5 and scikit-learn 0.23.2 libraries. Graphs are generated using the library matplotlib 3.3.4. For calculating the SVD, the *linalg.svd* function from the NumPy library is used, which wraps the *_gesdd* subroutine from the LAPACK Linear Algebra package.

Technical validation

The newly implemented SVD module has been validated on nine datasets from three unfolding kinetics and six temperature scanning experiments (Table S1). The performance was benchmarked against MATLAB R2019b (function *svd*) and KinTek Explorer 10.2.0 (3). For the latter, only the kinetics datasets were used since the tool does not support the analysis of temperature scanning experiments. The nine datasets used for validation can be found in the attached zip files (NAR_CalFitter2.0_SI_Supplementary_data_Validation folder).

The discrepancy for the three matrices U, Σ, and V and the difference between the reconstructions of M based on the first three components that explain most of the data variance were calculated. The reconstructed spectra were normalized by dividing by the maximal value. The discrepancy between CalFitter 2.0 and MATLAB results falls within the numerical precision error of the calculations, whilst it is slightly higher but still within 1% of the values compared to KinTek Explorer (Table S1).

Table S1. The absolute values of the differences between CalFitter 2.0 SVD module and MATLAB R2019b and KinTek 10.2.0 calculated based on the first three components.

	MATLAB R2019b (spectroscopy)				KinTek Explorer 10.2.0 (kinetics)*			
	Mean	St. dev.	Max	# points	Mean	St. dev.	Max	# points
Basis spectra (loadings)	1.36E-16	6.80E-16	2.97E-14	9345	2.26E-05	7.91E-05	9.67E-04	2853
Singular values	6.45E-16	4.83E-15	7.11E-14	240	2.56E-05	8.05E-05	3.26E-04	15
Signal components	7.54E-16	1.64E-15	1.60E-14	1209	3.21E-05	1.25E-04	1.17E-03	1080
Reconstructed spectra	2.29E-04	1.77E-04	5.00E-04	169041	N/A	N/A	N/A	N/A

* N/A = Not applicable; in cases where the raw numbers could not be extracted from KinTek Exlporer.

Use case: Unfolding of haloalkane dehalogenase DhaA

To demonstrate the application of the SVD analysis, CalFitter 2.0 was used to analyze the temperature-induced unfolding of haloalkane dehalogenase DhaA from soil bacterium *Rhodococcus rhodochrous*. This protein belongs to the large α/β hydrolase superfamily and is formed by the α/β core and the α-helical cap domain (PDB ID: 1CQW).

Materials and methods

DhaA was expressed in *Escherichia coli* BL21(DE3) cells and purified from the cell-free extract using affinity and size exclusion chromatography according to the published protocols (4). Its unfolding was monitored using differential scanning calorimetry (DSC), circular dichroism (CD), and fluorescence spectroscopy. Changes in heat capacity at two different scan rates (0.5 °C/min and 1 °C/min) were monitored using MicroCal VP-Capillary DSC System (Malvern, USA). Buffer-subtracted data were normalized to the protein concentration, followed by subtraction of a linear baseline connecting the pre- and post-transition signals. The fluorescence spectra of DhaA excited at 266 nm were collected at 300-450 nm range during temperature scanning experiments performed at different scan rates (0.3 °C/min, 1 °C/min, and 2 °C/min), or at fixed temperatures (40 °C, 45 °C, 50 °C, and 55 °C) over time using Uncle platform (Unchained Labs, USA). The CD spectra of DhaA were recorded in the far-UV range (185-260 nm) with a 1 nm bandwidth and 0.1 s integration time using Chirascan™ Circular Dichroism Spectrometer (Applied Photophysics, UK). During this experiment, the temperature was increased from 20 °C to 85 °C in 1 °C increments with 50 s equilibration and 10 s measurement times at each point, yielding approx. 1 °C/min scan rate. No hysteresis of the spectra occurred based on the comparison with the data collected at three wavelengths (195 nm, 210 nm, and 222 nm) during continuous heating of the sample at 1 °C/min. Each experiment was carried out in 50 mM potassium phosphate buffer pH 7.5. The protein concentrations used in CD, fluorescence, and DSC experiments were 0.17 $mg.ml^{-1}$, 0.5 $mg.ml^{-1}$, and 1 $mg.ml^{-1}$, respectively. Unfolding transitions were found to be independent of the protein concentration in the concentration range used in this study.

Dataset description and SVD analysis

All datasets used for the global analysis of DhaA unfolding can be found in the attached zip files (NAR_CalFitter2.0_SI_Supplementary_data_UseCase). The unfolding of DhaA occurs from approx. 35 °C to 65 °C and is scan rate dependent. The transition is associated with an endothermic calorimetric peak, a sharp decrease of fluorescence amplitude with a simultaneous red shift of the spectral maximum by approx. 10 nm (Figure S1A), and a loss of ellipticity at 195 nm and 225 nm (Figure S3A), based on the visual inspection of the DSC, fluorescence, and CD datasets, respectively. The time-dependent changes of the fluorescence spectra at fixed temperatures show similar trends as in the scanning experiments at varying rates according to the temperature of the experiment.

The spectra recorded at low temperatures, corresponding to the signal of the native state were fixed as a reference in all SVD analyses. The fluorescence datasets appear to be well approximated by the first two SVD components, which account for >98% of the observed variance (Figure S1B). Although the third singular value (green in Figure S1) is low compared to the first two, a closer inspection reveals that it has a distinct basis spectrum and its amplitude changes in a regular pattern with temperature, indicative of two transitions. The spectra reconstructed from the two or three components are visually almost indistinguishable, but the addition of the third component improves the residuals more significantly than the addition of the subsequent component (Figure S1C). Moreover, the autocorrelation coefficients of the third component derived from basis spectrum and the temperature dependence of its amplitude are 0.95 and 0.9, respectively. Therefore, we argue that the first three components are needed to fully explain the raw data and should be used in further global analysis. The rest of the components appear to be dominated by the experimental noise.

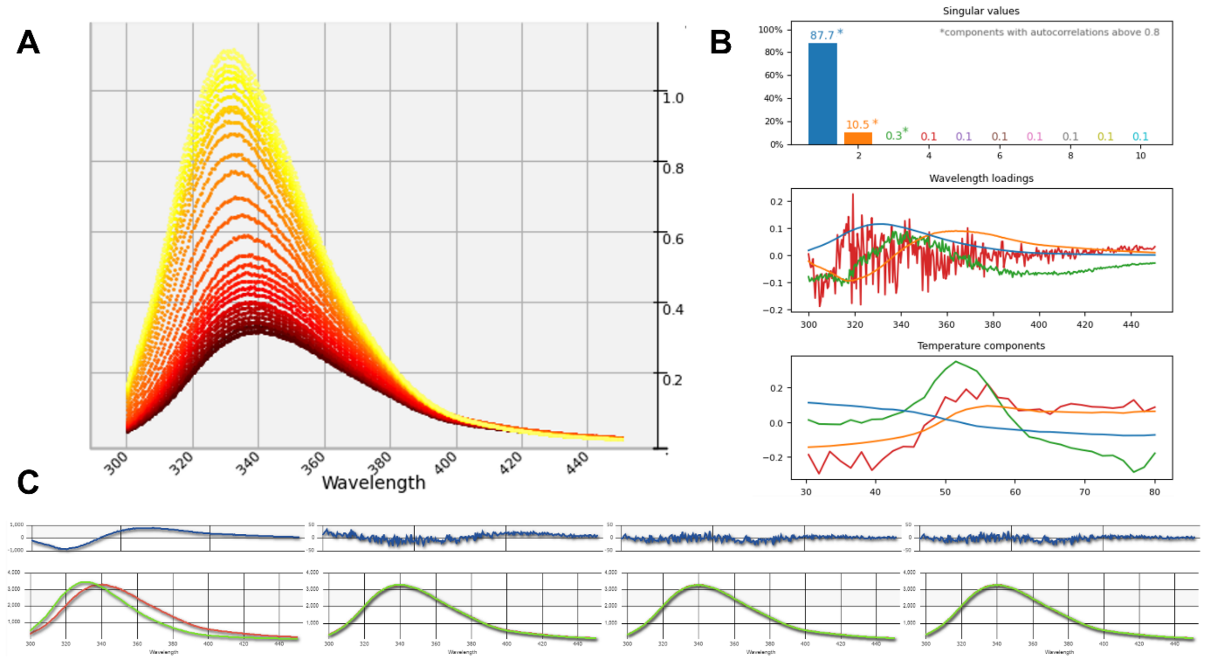

Figure S1: SVD analysis of the fluorescence spectroscopy data from a temperature scanning experiment recorded at the 1 °C/min scan rate. (A) Raw fluorescence spectra. Data show changes of DhaA fluorescence with increasing temperature (from yellow to dark red). **(B) Results of the SVD analysis.** Basis spectra (wavelength loadings) of the first four components are shown together with the

changes of their amplitudes with temperature (temperature components). **(C) Reconstruction of the raw spectrum of the denatured state** (red) based on the selected number of components (1-4 from left to right, depicted in green). The residuals between the original and reconstructed data are shown as blue lines in the corresponding graphs above.

The SVD analysis of the CD dataset reveals that the data are sufficiently explained by the first two components (Figure S3). Here, the judgment was made based on the visual inspection of the basis spectra and the temperature components rather than the singular values. Although the first two components account for only a 60 % of the observed variance, the basis spectra of the others seem to correspond to the experimental noise (Figure S3B). Indeed, excluding the wavelengths below 190 nm which contain the most experimental noise results in combined increase of 18% for the first two singular values. Although the basis spectra of the third and fourth component have regular patterns, their amplitudes do not change with increasing temperature and are therefore not considered for further global analysis. This decision is further supported by the autocorrelation of the basis spectra and the corresponding amplitude changes (Figure S2). The autocorrelation coefficient of the first two components of the CD Spectroscopy dataset calculated from both U (basis spectra) and V (temperature components) are close to 1, whereas the values oscillate around zero for the rest of the components, indicative of experimental noise.

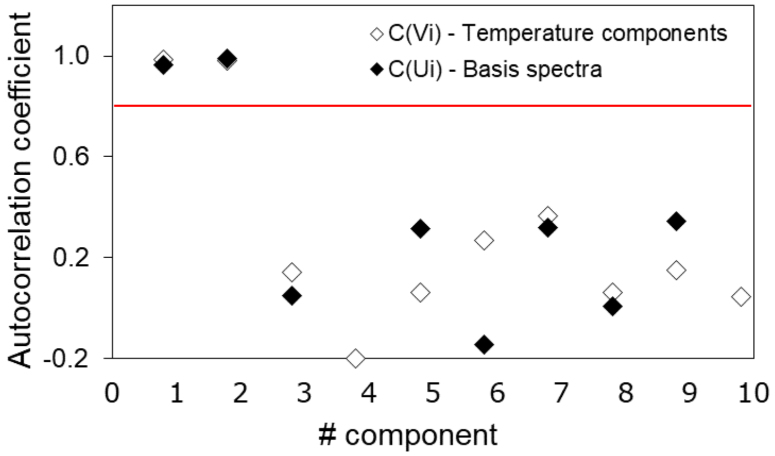

Figure S2: Autocorrelation coefficients for the basis spectra and the temperature changes of corresponding amplitudes for the first ten SVD components of the CD Spectroscopy dataset. The red line corresponds to the 0.8 threshold used in CalFitter to distinguish between the most probable significant components and experimental noise.

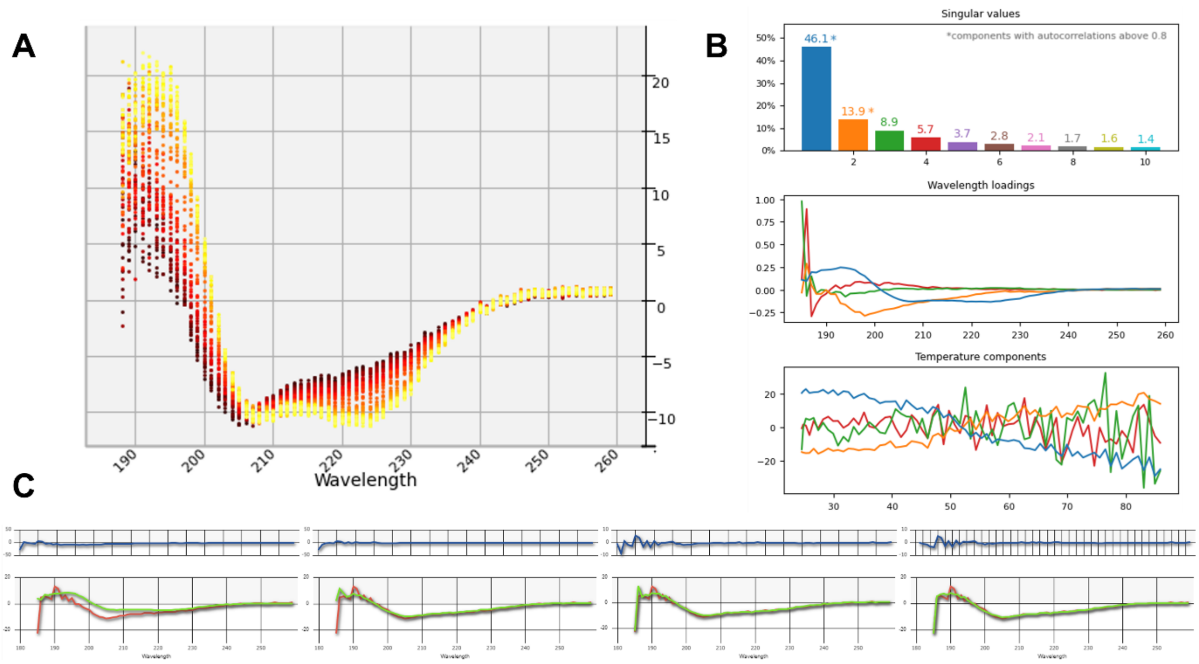

Figure S3: SVD analysis of the CD spectroscopy data from a temperature scanning experiment recorded at the 1 °C/min scan rate. (A) Raw CD spectra. Data show changes of DhaA ellipticity with increasing temperature (from yellow to dark red). **(B) Results of the SVD analysis**. Basis spectra (wavelength loadings) of the first four components are shown together with changes of their amplitudes with temperature (temperature components). **(C) Reconstruction of the raw spectrum** (red) based on the selected number of components (1-4 components left to right, depicted in green). The residuals between the original and reconstructed data are shown as blue lines in the corresponding graphs above.

Global fitting

All spectral datasets were processed using the SVD module of CalFitter 2.0, and their significant components were uploaded together with the DSC thermograms for subsequent global analysis of DhaA unfolding.

The first two most significant SVD components from the temperature scanning experiments can be fitted well to the two-state unfolding model (Figure S4A). However, the spectral reconstruction based on the modeled components deviates from the original data, especially in the middle of the apparent unfolding transition (bottom panel in Figure S4). Moreover, the two-state model fails to explain the amplitude changes of the third component, indicating that a more complex unfolding scenario must be considered. Indeed, the addition of an intermediate to the unfolding model results in a satisfactory fit to all three components of all three datasets (Figure S4B) and significantly improves the quality of the reconstructed spectra (bottom panel in Figure S4). Notably, the same fit of the data is achieved by two different models involving intermediate state, i.e., partially reversible, and fully irreversible, which differ in the modeling of the fraction of the intermediate state during temperature scan (Figure S4B and C). In the former, the intermediate accumulates to a considerable degree before it is irreversibly converted to the final denatured state ($k_{NI} \sim k_{IN} > k_{ID}$, middle panel in Figure S4B). This would indicate that the low singular value of the third component is caused by the spectral similarities between the highly populated intermediate and the other states. In the latter, the intermediate never accumulates to a significant amount since the rate of the second step is faster than that of the first ($k_{NI} < k_{ID}$, middle panel in

Figure S4C). In this case, low singular value of the third component could be directly correlated with the low population of the intermediate state.

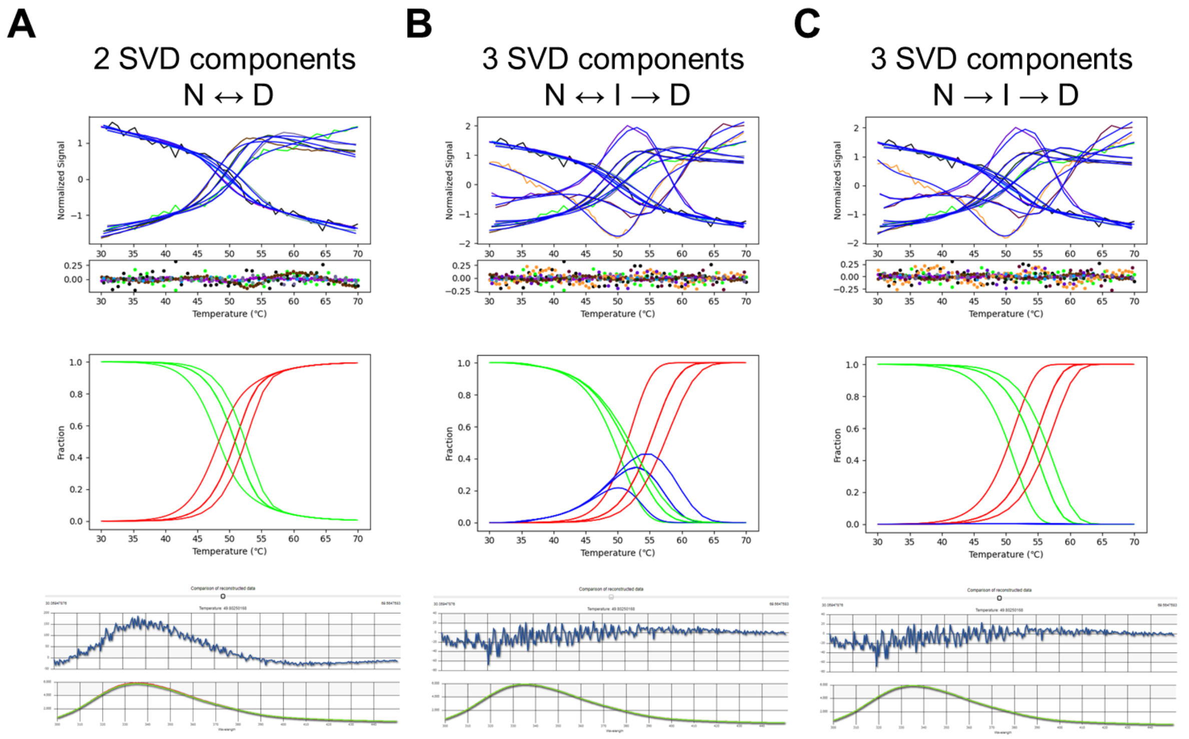

Figure S4: Global fitting of different number of SVD components from Spectroscopy SVD datasets recorded at 0.3 °C/min, 1 °C/min, and 2 °C/min scan rates to different unfolding models. A) First two components fitted to the two-state reversible model. **B)** First three components fitted to the three-state partially reversible model. **C)** First three components fitted to the three-state irreversible model. **Top**: Results of the global fitting (blue lines) and the corresponding graphs of the residuals. **Middle**: Fraction of states based on the modeled parameters. Native, intermediate, and denatured states are shown in green, blue, and red, respectively. **Bottom:** Reconstruction of the original spectrum at 50°C (red lines) using the fitted SVD components (green lines). The corresponding residuals are shown in dark blue in the graph above.

To resolve which model better characterize DhaA unfolding, Kinetics SVD and calorimetric data were fitted together with the SVD components from the temperature scanning experiments. The calorimetric thermograms contain a high temperature “shoulder” peak which provide another evidence in favor of the three-state model. Moreover, the third components from the Kinetics SVD datasets show biphasic behavior, i.e., involve two distinct kinetics phases with opposing amplitudes. Furthermore, the partial reversibility of the unfolding has been validated by the observation that when the DhaA is cooled down from the temperature just after the transition (~60 °C), only a fraction of the native state is recovered, as judged by the re-heating scan (data not shown). Indeed, all datasets can be globally fitted to the three-state partially reversible model of unfolding (Figure S5).

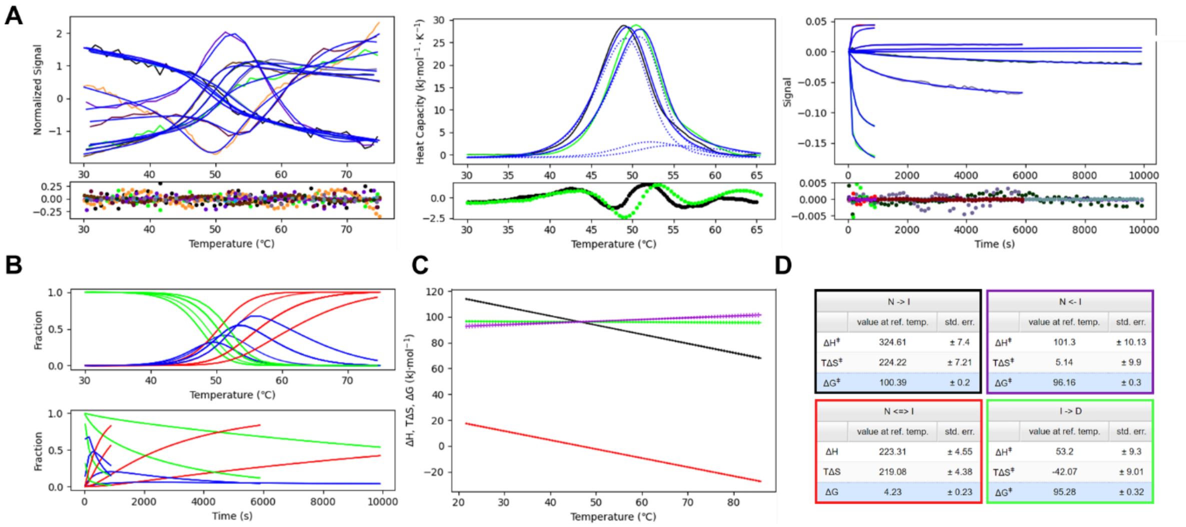

Figure S5: Global fitting of the DhaA unfolding data to the three-state partially reversible model of unfolding. A) Global fit of the Spectroscopy SVD (left), Calorimetry (middle), and Kinetics SVD datasets to the model (blue lines). **B)** Fraction of states based on the model as a function of temperature (top), or time (bottom). Native, intermediate, and denatured states are depicted by green, blue, and red lines, respectively. **C)** Gibbs free energy of unfolding (ΔG_0, black), and the Gibbs activation energies ($\Delta G^{\ddagger}$, color coded according to D). **D)** The energy parameters at 40°C derived from the curves in C (with the same color coding).

All datasets are modeled well judged by the residual graphs (Figure S5A). The goodness-of-fit of the was further evaluated by another novel feature of CalFitter 2.0 - reconstruction of the original data based on the fitted SVD component amplitudes, described in the initial part of this supplementary. The model spectra closely follow those constructed from the raw data in most cases (Figure S6). The fit deviates slightly from the calorimetric curves mainly due to the uncertainty in dissecting the calorimetric enthalpy between the two steps. The barriers separating the intermediate from the native and the irreversibly denatured states appear to have very similar energies (Figure S5C). Consequently, the parameters describing these steps compensate each other during fitting, prohibiting their precise resolution. However, the energy barrier of the first unfolding step (black in Figure S5C) is modeled very robustly and provides a suitable quantitative measure describing the thermostability of DhaA (black in Figure S5C and D).

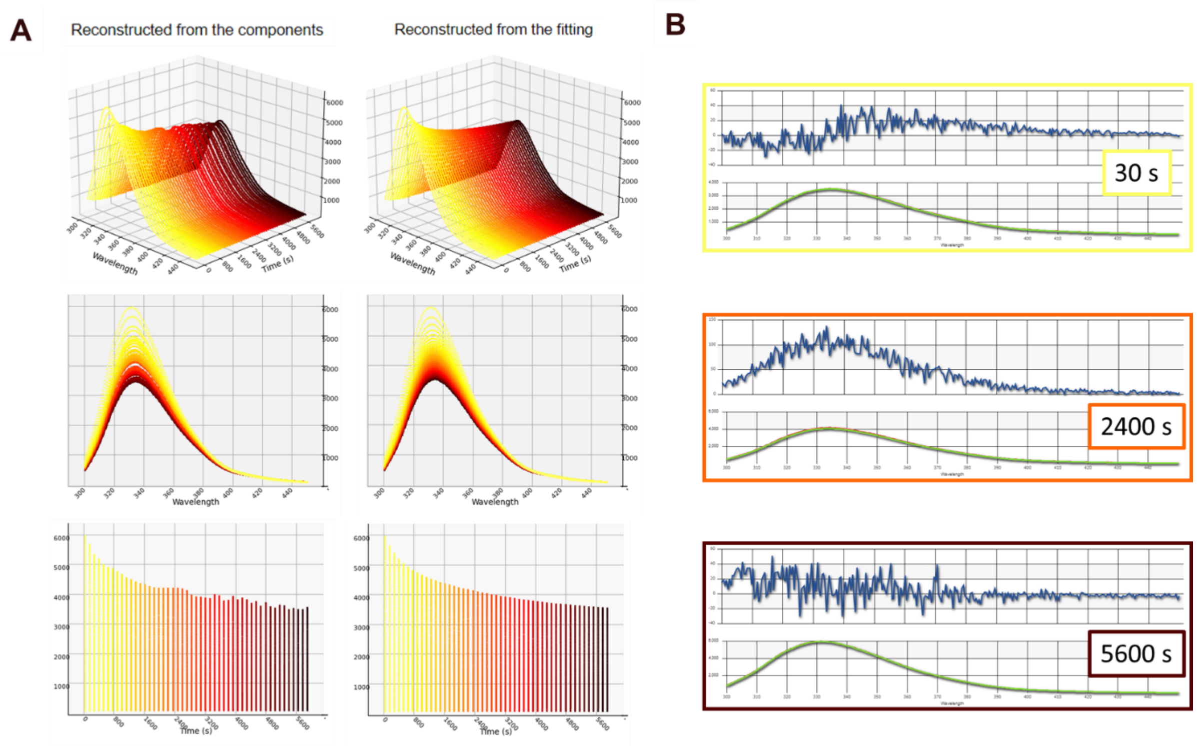

Figure S6: Spectral reconstruction of the CD spectroscopic dataset based on the modelled parameters. (A) Side-by-side comparison of the data reconstructed from the two components selected during initial SVD analysis (left), and those reconstructed from the components calculated based on the three-state partially reversible model (right). **(B) Dissection of the spectra at three different time points.** The difference between the spectra reconstructed from the initial (red) and modeled (green) components is shown in the residual graphs above the corresponding spectra.

The case study shown here highlights the strengths of the SVD analysis in the resolution of spectral fingerprints of the lowly populated states from the spectroscopic data. Simultaneously, it clearly demonstrates the importance of using different experimental techniques combined with the global fitting of the resulting data for the quantitative description of protein thermostability.

References

1. Eckart,C. and Young,G. (1936) The approximation of one matrix by another of lower rank. *Psychometrika*, **1**, 211–218.

2. Mazurenko,S., Stourac,J., Kunka,A., Nedeljković,S., Bednar,D., Prokop,Z. and Damborsky,J. (2018) CalFitter: a web server for analysis of protein thermal denaturation data. *Nucleic Acids Research*, **46**, W344–W349.

3. Johnson,K.A., Simpson,Z.B. and Blom,T. (2009) Global Kinetic Explorer: A new computer program for dynamic simulation and fitting of kinetic data. *Analytical Biochemistry*, **387**, 20–29.

4. Markova,K., Chmelova,K., Marques,S.M., Carpentier,P., Bednar,D., Damborsky,J. and Marek,M. (2020) Decoding the intricate network of molecular interactions of a hyperstable engineered biocatalyst. *Chemical Science*, **11**, 11162–11178.
